# Mycoplasma pneumoniae-Induced Erythema Multiforme in an 11-Year-Old Boy: Diagnostic and Therapeutic Challenges

**DOI:** 10.7759/cureus.79944

**Published:** 2025-03-03

**Authors:** Hamdah T Kalantar, Suneel Pooboni

**Affiliations:** 1 Medicine, Mohammed Bin Rashid University of Medicine and Health Sciences (MBRU), Dubai, ARE; 2 Pediatrics, Mediclinic City Hospital, Dubai, ARE

**Keywords:** erythema multiforme, hypersensitivity reaction, immune-mediated, mucocutaneous rash, mycoplasma pneumoniae, steven johnson syndrome

## Abstract

*Mycoplasma pneumoniae* is a causative agent of respiratory tract infections that can also trigger immune-mediated complications such as erythema multiforme (EM), presenting with characteristic target-like skin lesions. Recognizing *M. pneumoniae* as a potential cause of EM is crucial for accurate diagnosis and timely intervention.

We present the case of an 11-year-old boy hospitalized with *M. pneumoniae *infection followed by severe EM. Initially presenting with high fever, oral ulcers, and a characteristic rash on his extremities, he was misdiagnosed with pharyngotonsillitis and herpetic gingivostomatitis. His symptoms persisted despite treatment until a positive polymerase chain reaction (PCR) confirmed an *M. pneumoniae* infection. By day 6, the rash had evolved into target lesions, leading to a revised diagnosis of EM. The patient was treated with IV azithromycin, corticosteroids, and IV immunoglobulin (IVIg), which resulted in significant clinical improvement.

Recognizing *M. pneumoniae* as a potential cause of EM is essential for accurate and prompt diagnosis. Timely identification and a multidisciplinary treatment approach, including appropriate antimicrobial therapy and supportive care, can significantly enhance patient outcomes.

## Introduction

*Mycoplasma pneumoniae* is a bacterium that causes infections in the upper respiratory tract and atypical pneumonia [1]. *M. pneumoniae* infection can affect the skin and may lead to mucosal involvement in up to 25% of cases, potentially leading to erythema multiforme (EM) and Stevens-Johnson syndrome (SJS)/toxic epidermal necrolysis (TEN) [2]. *M. pneumoniae* is more prevalent among the population under 40. *M. pneumoniae* infections can occur year-round, but community outbreaks occur every three to seven years. Clinical manifestations of *M. pneumoniae *include mild symptoms such as fever, cough, sore throat, malaise, and headache, while EM presents with target-like lesions on the skin, often accompanied by mucosal involvement such as oral ulcers and conjunctivitis. Ocular involvement occurs in approximately 30% of pediatric cases. In most cases, symptoms are mild, but in severe cases, *M. pneumoniae *can cause critical complications: acute pneumonia, encephalitis, renal impairment, and hemolytic anemia, especially in the elderly or immunocompromised population. Transmission occurs through respiratory droplets from coughing or sneezing or direct contact with infected nasal or throat discharges, or it may happen indirectly through contaminated articles. The period of incubation ranges from two to three weeks, where mild cases resolve spontaneously most of the time, but if they become severe, antibiotic treatment is prescribed [1]. However, 5% to 10% of infected patients with *M. pneumoniae* may develop atypical pneumonia [1].

*M. pneumoniae* is also known to trigger various immune-mediated extrapulmonary manifestations, including EM, an acute hypersensitivity reaction affecting the skin and mucosal surfaces. EM is classified into two subtypes: EM minor, which primarily involves localized skin lesions, and EM major, which presents with significant mucosal involvement and may resemble severe conditions like SJS. Recognizing the spectrum of EM severity is essential for accurate diagnosis and appropriate management.

EM, on the other hand, is an acute, self-limiting skin condition; it is occasionally recurring and is categorized as a type IV hypersensitivity reaction [3]. EM is mostly triggered by infections, medications, and other factors. Disease severity spans a spectrum as target lesions evolve over 72 hours, with EM minor affecting the extremities, and they heal within a week, while acute EM conditions involve mucous membrane formation and epidermal detachment. Clinical significance of EM is it shows papular, bullous, and necrotic lesions on skin and mucosal membranes, typically necessitates medication in approximately 50% of cases to manage its recurrent nature while *M. pneumoniae* is also significant cause of community-acquired pneumonia (CAP) in children and accounts for 10-40% of cases requiring hospitalization [4].

Traditional methods for diagnosing *M. pneumoniae* infections, such as serology and culture, have limitations due to delayed results and reduced sensitivity. Polymerase chain reaction (PCR) testing has become the preferred diagnostic approach because it provides rapid, highly sensitive detection of *M. pneumoniae* DNA, facilitating early and accurate diagnosis. PCR testing is particularly useful in distinguishing *M. pneumoniae* from other infectious causes of EM, enabling timely and targeted treatment.

We selected this case because it presents an atypical presentation of *M. pneumoniae*-induced EM in a child manifesting with severe oral ulcers, widespread rash, and mucosal involvement. Our patient presented with systematic symptoms, showing the rarity and complexity of this disease, and this case contributes to understanding differential diagnosis and management strategies in the younger pediatric population with *M. pneumoniae*-induced EM.

## Case presentation

An 11-year-old boy presented with persistent oral ulcers, a rash on his hands and feet, high-grade fever, lip swelling, and painful swallowing. Physical examination revealed dehydration, alertness, and orientation without distress. His symptoms evolved over several days, necessitating careful monitoring of disease progression.

On March 8th, he was brought to the emergency room with a fever, ear and throat congestion, and dehydration and was initially treated for pharyngotonsillitis.

**Figure 1:**
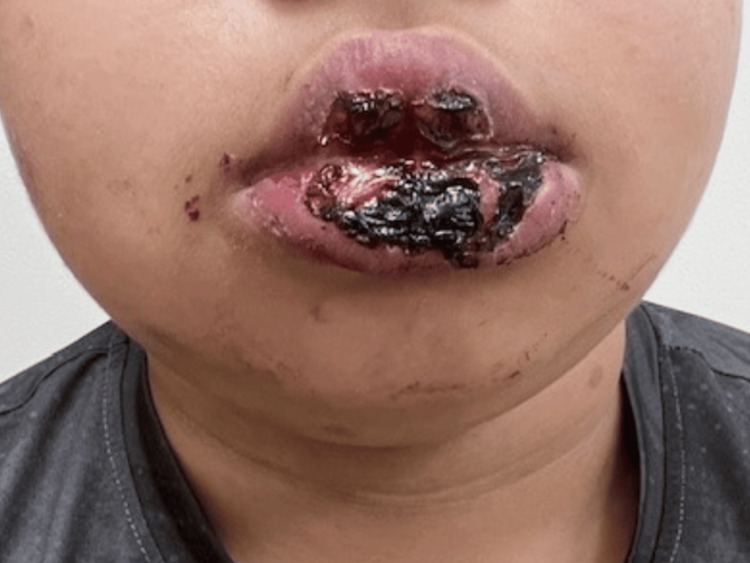
Oral Mucosal Lesions in Erythema Multiforme: Erosions and Ulcerations

On March 9th, oral ulcers appeared as small lesions, which progressively ulcerated, causing significant pain and bleeding. These ulcers persisted and enlarged, leading to lip swelling, painful swallowing, and poor oral intake (Figure 1). The patient had no significant past medical history, no previous episodes of similar ulcers, and no known family history of genetic or vascular diseases.

On March 10th, despite ongoing symptomatic treatment, the fever remained intermittent, with a peak temperature of 38.6°C, temporarily alleviated by antipyretics. The rash on his extremities, initially erythematous, evolved in distribution and morphology. A skin rash developed over the hands and feet two days prior to admission, initially presenting as erythematous plaques with mild pruritus. Satellite lesions were observed on both hands and feet, with additional lesions on the back. The rash persisted throughout the day without known exacerbating or relieving factors.

On March 11th, the patient was misdiagnosed with herpetic gingivostomatitis due to overlapping symptoms, and treatment with IV ceftriaxone, IV acyclovir, IV chlorohistol, IV paracetamol, and oral ibuprofen was initiated. However, he showed no clinical improvement. PCR testing confirmed an *M. pneumoniae* infection, prompting the addition of IV azithromycin.

On March 12th, despite targeted antibiotic therapy, his condition worsened, necessitating transfer to the Pediatric Intensive Care Unit (PICU).

On March 14th, the rash evolved into well-defined target lesions, a characteristic of EM.

Treatment response over time

Despite broad-spectrum antibiotics, the patient did not respond adequately to initial antimicrobial and antiviral therapy. The addition of IV azithromycin led to partial improvement, but significant mucocutaneous symptoms persisted. Given the progression of mucosal involvement and systemic inflammation, corticosteroids and IV immunoglobulin (IVIg) were introduced, leading to significant clinical improvement over several days.

By day 6 post-admission, the fever had subsided, and the oral ulcers showed signs of healing. The skin lesions began resolving, with reduced erythema and swelling. The patient’s hydration and nutritional status improved following IV fluid support and pain management. Serial monitoring of inflammatory markers, including CRP, showed a downward trend, correlating with clinical improvement.

This case highlights the importance of a structured symptom timeline and close monitoring of treatment response in *M. pneumoniae*-induced EM.

Findings

In Table 1, the patient's lab results revealed several abnormalities. The elevated CRP level of 145 mg/L is significantly above the normal range (usually <10 mg/L), indicating acute inflammation or infection. The white blood cell (WBC) count was low at 3.24 × 10^3/uL (normal range: 4.5-11.0 x 10^3/uL), suggestive of leukopenia, which can occur in severe infections or inflammatory conditions. Among the WBC, the differential count showed neutrophils at 7.49 x 10^3/uL (upper normal range but relatively high given the low total WBC count), lymphocytes at 1.01 x 10^3/uL (normal range: 1.5-3.5 x 10^3/uL), and monocytes at 1.4 x 10^3/uL (normal range: 0.2-0.8 x 10^3/uL), indicating a relative neutrophilia and monocytosis, which are responses to infection. Eosinophils at 0.049 x 10^3/uL were within the lower end of the normal range (0.05-0.5 x 10^3/uL), which is slightly low. The hemoglobin level of 11.6 g/dL is slightly below the normal range (13.5-17.5 g/dL for males), indicating mild anemia, potentially due to the chronic disease process or inflammation. The mean corpuscular volume (MCV) of 77.1 fL (normal range: 80-100 fL) and mean corpuscular hemoglobin (MCH) of 25.3 pg (normal range: 27-33 pg) suggest microcytic, hypochromic anemia. Albumin was slightly low at 33 g/L (normal range: 35-50 g/L), indicating possible inflammation or poor nutritional status. Bicarbonate was low at 19.7 mmol/L (normal range: 22-29 mmol/L), suggesting a mild metabolic acidosis or compensatory response to respiratory alkalosis.

**Table 1:** Summary of Clinical Findings and Laboratory Investigations HEENT: head, ears, eyes, nose, and throat; PCR: polymerase chain reaction; PICU: pediatric intensive care unit; GCS: Glasgow Coma Scale

Finding	Value	Normal Range	Notes
Temperature	38.6°C	36.5-37.5°C	Within normal limits for PICU setting, afebrile at time of examination.
Heart Rate	97 bt/min	60-100 bt/min	Within normal limits for age.
Systolic Blood Pressure	111 mmHg	90-120 mmHg	Within normal limits.
Diastolic Blood Pressure	80 mmHg	60-80 mmHg	On the higher side of normal but within acceptable limits.
Respiratory Rate	18 br/min	12-20 br/min	Within normal limits.
SPO_2_	100%	95-100%	Normal oxygen saturation.
Height	162 cm	Not applicable	Normal for age.
Weight	72.9 kg	Not applicable	Normal for height, but BMI indicates overweight.
Body Mass Index (BMI)	27.78 kg/m²	18.5-24.9 kg/m²	Indicates overweight status.
General Appearance	Dehydrated, scared	-	Shows signs of dehydration and distress.
HEENT	Lip swelling, ulcers, ear and throat congestion	-	Significant abnormalities including active bleeding ulcers and congestion.
Respiratory	Mild wheezing	-	Wheezing indicates possible respiratory distress or infection.
Cardiovascular	S1 and S2 heard, distal pulses palpable, no edema	-	Within normal limits.
Abdominal	Soft, non-tender, normal bowel sounds		No abnormalities detected.
Skin	Erythematous plaques (target lesions)	-	Presence of erythematous target lesions and satellite lesions.
Neurological	GCS 15/15, normal coordination	GCS 15/15, normal coordination	No neurological deficits detected.
Hemoglobin	11.6 g/dL	13.5-17.5 g/dL (males)	Indicates mild anemia.
White Cell Count (WBC)	3.24 10^3/uL	4.0-11.0 10^3/uL	Low WBC count, possibly due to infection or immune response.
Neutrophils	7.49 10^3/uL	2.0-7.0 10^3/uL	Elevated, indicating acute infection or inflammation.
Lymphocytes	1.01 10^3/uL	1.5-4.0 10^3/uL	Low lymphocyte count, possibly due to infection or stress.
Monocytes	1.4 10^3/uL	0.2-1.0 10^3/uL	Elevated, indicating chronic infection or inflammation.
Platelet Count	257 10^3/uL	150-450 10^3/uL	Within normal limits.
Red Blood Cell Count	4.33 10^6/uL	4.5-5.9 10^6/uL	Slightly low but within acceptable limits.
Mean Platelet Volume (MPV)	7.7 fL	8.0-12.0 fL	Low, indicating possible bone marrow suppression or other hematological issues.
Mean Corpuscular Volume (MCV)	77.1 fL	80-100 fL	Low, indicating microcytic anemia.
Mean Corpuscular Hemoglobin (MCH)	25.3 pg	27-34 pg	Low, consistent with microcytic anemia.
C-reactive Protein (CRP)	145 mg/L	<5 mg/L	Significantly elevated, indicating severe inflammation or infection.
Albumin	33 g/L	35-50 g/L	Slightly low, could indicate chronic illness or malnutrition.
Total Bilirubin	5.07 umol/L	1.2-3.0 umol/L	Mildly elevated, possible liver dysfunction or hemolysis.
Direct Bilirubin	2.02 umol/L	<1.7 umol/L	Elevated, possible liver dysfunction or bile duct obstruction.
Alkaline Phosphatase (ALP)	220 U/L	44-147 U/L	Elevated, indicating liver or bone disease.
Alanine Aminotransferase (ALT)	14.3 U/L	7-56 U/L	Within normal limits.
Aspartate Aminotransferase (AST)	24.8 U/L	10-40 U/L	Within normal limits.
Total Protein	69.9 g/L	60-80 g/L	Within normal limits.
Sodium	140.5 mmol/L	135-145 mmol/L	Within normal limits.
Potassium	4.43 mmol/L	3.5-5.0 mmol/L	Within normal limits.
Chloride	106.2 mmol/L	98-107 mmol/L	Within normal limits.
Bicarbonate	19.7 mmol/L	22-29 mmol/L	Low, indicating possible metabolic acidosis.
Blood Culture	No growth	-	No bacterial growth detected.
Measles and Rubella PCR	Negative	-	No viral infection detected.
Streptococcus Group A Antigen Test	Negative	-	No *Streptococcus* infection detected.
Mycoplasma Pneumonia Antibody IgM	Positive	-	Indicates acute *Mycoplasma pneumoniae* infection.
Herpes Simplex Virus PCR	Negative	-	No Herpes simplex virus infection detected.
Respiratory Panel	Positive for human rhinovirus/enterovirus	-	Indicates concurrent viral infection.

Conversely, other lab values were within normal limits, such as liver function tests (total bilirubin 5.07 umol/L, direct bilirubin 2.02 umol/L, alanine transaminase (ALT) 14.3 U/L, aspartate transaminase (AST) 24.8 U/L), and the electrolyte panel (sodium 140.5 mmol/L, potassium 4.43 mmol/L, chloride 106.2 mmol/L). Alkaline phosphatase (ALP) at 220 U/L is within the upper range of normal but not alarmingly high (normal range: 44-147 U/L), often varying with age and growth spurts in children. The PCR test confirmed the presence of *M. pneumoniae* by detecting its specific DNA, correlating it with the patient's clinical symptoms, and guiding the use of appropriate antibiotic therapy (IV azithromycin). Other tests, including blood cultures, measles, and rubella PCR, and Streptococcus Group A antigen test, were negative, helping rule out other potential infectious agents.

Diagnosis

A positive IgM antibody test confirmed a recent or acute *M. pneumoniae* infection.

Treatment

The patient’s management involved a combination of antimicrobial therapy, anti-inflammatory agents, immune modulation, and supportive care tailored to the severity of SJS. Given the confirmed *M. pneumoniae* infection and suspected viral co-infection, IV ceftriaxone and azithromycin were administered, along with IV acyclovir for potential herpetic involvement. To control inflammation and mitigate immune-mediated damage, corticosteroids and IVIg were utilized. Pain and fever management were achieved with IV paracetamol and oral ibuprofen, while antihistamines such as IV diphenhydramine and chlorohistol helped alleviate allergic symptoms. Hydration and electrolyte balance were maintained through IV fluids (100 mL of D5NS), and topical anesthetics were applied for the symptomatic relief of oral ulcers. He did not require any parenteral or enteral nutrition. Table 2 summarizes the detailed treatment regimen administered throughout hospitalization.

**Table 2:** Summary of Treatment Regimen for Erythema Multiforme

Drug Name	Recommended Dosage	Route	Function
IV ceftriaxone	1000 mg twice daily for 4 days	IV	Broad-spectrum antibiotic used to treat bacterial infections such as pharyngo-tonsillitis.
IV acyclovir	325 mg every 8 hours for 5 days	IV	Antiviral medication used to treat herpetic gingivostomatitis.
IV chlorohistol (diphenhydramine HCl)	50 mg over 30 min for 5 days	IV	Antihistamine to reduce allergy symptoms like rash and itching.
IV paracetamol	650 mg over 20 min PRN	IV	Antipyretic and analgesic used to reduce fever and alleviate pain.
Oral brufen (ibuprofen)	5-10 mg every 6 to 8 hours, 5 days	Oral	Nonsteroidal anti-inflammatory drug (NSAID) used for pain relief and to reduce inflammation.
IV azithromycin	500 mg daily for 3 days	IV	Antibiotic used to treat infections caused by *Mycoplasma pneumoniae*.
IVIg	30 g over 6 hours, over 3 days	IV	Used to modulate the immune system and reduce severe inflammation.
Hydrocortisone sodium succinate	100 mg over 20 min	IV	Corticosteroid to reduce severe inflammation and immune response.
IV diphenhydramine HCl	50 mg over 30 min	IV	Antihistamine to treat allergic reactions and symptoms like itching and rash.
Dextrose 5% and 0.9% saline	100 mL per hour	IV	IV fluid for hydration and to maintain electrolyte balance.
Lidocaine HCl/aminoacridine HCl gel	0.66%-0.05% gel every 8 hours	Topical	Topical anesthetic and antiseptic for pain relief and to reduce inflammation of oral ulcers.

Monitoring

The patient’s temperature, level of consciousness, heart rate, blood pressure, respiratory rate, and oxygen saturation were monitored in PICU to detect the progression rate. To control the high temperature, antipyretics and IV paracetamol were given. We documented oral ulcers and skin lesions every 12 hours, and changes in size, appearance, or new lesions. Hydration status was regulated through physical signs such as skin turgor, mucous membrane moisture, and urine output, and IV fluids were adjusted in a timely manner. Regular complete blood count (CBC) tests were conducted to monitor WBCs, which helps to detect inflammation and infection. Hemoglobin and hematocrit levels ensured stability, while CRP (initially 145 mg/L) was checked every 48 hours post-treatment. Electrolytes and renal function were monitored closely.

Antibiotics and antiviral drug doses and frequency of IV ceftriaxone, IV acyclovir, and IV azithromycin were strictly followed, with adjustments made based on renal function and the patient’s response to treatment. After azithromycin was added to treatment, its effectiveness was judged by clinical improvement and reduction in symptoms rather than serum levels. IVIg and hydrocortisone were given to the patient, and its efficiency was monitored through improvements in mucocutaneous lesions and overall inflammation. Optimal hydration and nutrition were managed with IV fluids, and the rate of dextrose 5% and 0.9% saline infusion (100 mL/hour) were adjusted. We monitored fluid balance by measuring input and output every eight hours, adjusting IV fluid rates as necessary to maintain hydration and electrolyte balance. The patient showed improvement with the administered treatment regimen. We recommended consultation with a dermatologist.

## Discussion

Besides causing respiratory infection, *M. pneumoniae* - implicated in up to 40% of atypical pneumonia cases - can also manifest with extrapulmonary complications like mucocutaneous eruptions, including EM, SJS, and *M. pneumoniae*-induced rash and mucositis (MIRM). The exact incidence of EM caused by *M. pneumoniae* is not well documented [5]. However, studies suggest that up to 25% of infected patients may present with cutaneous involvement, highlighting the need for increased clinical awareness [6]. Identification of *M. pneumoniae* as the causative agent is often challenging due to symptom overlap with conditions such as Kawasaki disease, Sweet syndrome, Henoch-Schönlein purpura, Raynaud disease, and viral exanthems [5].

EM is classified into minor and major subtypes. EM minor presents with localized skin lesions, typically resolving within a week, whereas EM major is characterized by significant mucosal involvement and epidermal detachment, sometimes mimicking severe disorders like SJS [6]. The differential diagnosis is crucial in distinguishing EM from conditions such as SJS, TEN, and viral infections, which may present with similar mucocutaneous findings [6].

Molecular mechanisms and immune response

*M. pneumoniae*-induced EM is primarily immune-mediated rather than a direct bacterial invasion. Due to its lack of a cell wall, *M. pneumoniae *evades traditional antibiotics and triggers a host immune response, leading to tissue damage [7]. The pathogen activates the production of proinflammatory cytokines such as interleukin 1-alpha (IL-1α), IL-1β, IL-6, IL-12, and IL-17, which in turn stimulate CD8+ T cells, tumor necrosis factor-alpha (TNF-α), and interferon-gamma (IFN-γ), contributing to epidermal apoptosis and mucosal involvement [8].

A key pathogenic factor is the P1 adhesin gene (mpn141), which enables bacterial adherence to respiratory epithelial cells, facilitating colonization and immune activation [9]. Additionally, the CARDS toxin (mpn372) plays a critical role in respiratory distress by modulating immune responses and inducing epithelial damage [10]. The antigenic variation of vlp genes (vlpA, vlpB, vlpC) enables immune evasion, complicating host defenses against infection [11].

Although this case supports the role of cytokine activation in disease severity, comparing molecular findings with other documented cases of *M. pneumoniae*-induced EM would enhance our understanding of its pathogenesis. Recent studies indicate that microRNA regulation, particularly miRNA-492, influences cytokine secretion in mononuclear macrophages, further affecting disease severity [12].

Research suggested the bacterium's ability to adhere, damage respiratory epithelial cells, evade immune responses, and induce inflammation, which is central to pathogenic mechanisms [12]. Critical to this process is the P1 adhesin gene (mpn141), which encodes the P1 protein that enables *M. pneumoniae* to attach to host cells, facilitating colonization. Additionally, the CARDS toxin gene (mpn372) produces an exotoxin that contributes to respiratory distress and inflammation by damaging cells and modulating the immune response. The variable adherence-associated antigen genes vlpA, vlpB, and vlpC enable the bacterium to evade the immune system through antigenic variation. Msa1 and msa2 genes that encode surface proteins enhance adhesion at greater extant and allow immune evasion, ultimately aiding in the persistence and pathogenicity of *M. pneumoniae* [12].

Adhesion and colonization are initiated by the interaction of the P1 adhesin with sialic acid-containing receptors on epithelial cells. This enable *M. pneumoniae *to resist mucociliary clearance. Vlp genes continuously alter surface antigens and allow immune evasion, preventing effective immune recognition and clearance. Respiratory distress and inflammation is worsen by CARD toxins, disrupting epithelial function and integrity while modulating immune responses. Pro-inflammatory cytokines are released by infection, including TNF-α, IL-6, and IL-18, mediating inflammation and clinical symptoms [12].

Other contributing factors include the host immune response, which, while critical in controlling the infection, can cause immunopathology when excessively activated. MicroRNA regulation, specifically miRNA-492, influences the immune response by regulating cytokine secretion in mononuclear macrophages, affecting disease severity. Environmental factors such as crowded living conditions, poor ventilation, and seasonal variations also play a role in the transmission and incidence of infections. Genetic predisposition, particularly in immune response-related genes, can impact susceptibility and disease severity. Understanding these genetic and molecular mechanisms is essential for developing targeted therapies and improving clinical outcomes in pediatric populations affected by *M. pneumoniae* [12].

While identifying *M. pneumoniae,* several differential diagnoses were considered due to overlapping symptoms, including oral ulcers, rash, high-grade fever, lip swelling, and painful swallowing, which increased the risk of misdiagnosis. Initially, herpetic gingivostomatitis was suspected, given its characteristic oral ulcers and fever; however, a PCR test for herpes simplex virus was negative. Attention then shifted to SJS due to the development of target lesions and mucocutaneous involvement, but the patient did not exhibit the full spectrum of systemic manifestations typically seen in SJS. Other differential diagnoses included hand-foot-and-mouth disease (HFMD), caused by enteroviruses, given the rash on the hands and feet and oral ulcers. However, the severity and persistence of symptoms were atypical for HFMD. Kawasaki disease was also considered due to fever, mucocutaneous symptoms, and rash, but the absence of key diagnostic features such as conjunctival injection, extremity changes, and lymphadenopathy made this less likely. Ultimately, *M. pneumoniae *infection was confirmed with a positive IgM antibody test and a respiratory panel showing human rhinovirus/enterovirus. The diagnosis was further supported by the patient’s response to azithromycin and the exclusion of other potential causes through targeted testing and clinical evaluation.

*M. pneumoniae*-induced EM has been documented in pediatric cases, but the severity and extent of mucosal involvement vary. Studies suggest that *M. pneumoniae *accounts for up to 25% of EM cases, with mucosal involvement observed in approximately 70% of affected patients. Compared to other reports, our case aligns with the established presentation of *M. pneumoniae*-induced EM but is notable for its initial misdiagnosis and progression despite early antibiotic treatment. Similar cases have described atypical presentations, such as a nine-year-old with predominant ocular involvement, including bilateral conjunctivitis and severe photophobia, and a 14-year-old initially suspected of having HFMD due to extensive palmar and plantar desquamation, later confirmed as *M. pneumoniae*-induced EM via PCR testing. Some cases have also mimicked Kawasaki disease, leading to further diagnostic confusion. A large case series by Meyer Sauteur et al. reviewed 30 pediatric cases of *M. pneumoniae*-induced EM and found that nearly 50% were initially misdiagnosed, most commonly as viral exanthems or SJS [13]. Compared to these cases, our patient demonstrated classic mucocutaneous involvement but without significant systemic manifestations such as hemodynamic instability or multiorgan dysfunction. This case reinforces the importance of clinical vigilance in distinguishing *M. pneumoniae*-induced EM from other severe cutaneous drug reactions and infectious exanthems.

Diagnostic challenges and treatment considerations

EM is possibly differentially diagnosed from SJS, TEN, viral infections, drug reactions, autoimmune diseases, allergic reactions, or infectious diseases based on clinical presentation and associated symptoms [14]. Target or iris lesions are hallmarks of EM, including the *M. pneumoniae*-induced subtype. These lesions manifest as round erythematous papules or plaques with central clearing, resembling a target or bull's-eye appearance [15]. They often develop on the extremities and may spread to other areas of the body. *M. pneumoniae*-induced EM sometimes develops mucous membranes, presenting as oral ulcers, lip swelling, and painful swallowing. Mucosal involvement distinguishes EM from other cutaneous eruptions and may contribute to the severity of the disease [16]. *M. pneumoniae*-induced EM may also exhibit respiratory symptoms like cough, sore throat, and congestion, often preceding or coinciding with skin lesions and EM [17], and when it triggers EM, sometimes patients may need diverse treatment strategies beyond azithromycin. Other effective antibiotic options are doxycycline, levofloxacin, moxifloxacin, and minocycline. Minocycline has been shown to be effective in treating pediatric *M. pneumoniae* infections by shortening the time to defervescence (TTD) and improving clinical outcomes. It acts by targeting the 30S ribosomal subunit and inhibiting bacterial protein synthesis. However, macrolides such as azithromycin remain the first-line treatment due to their favorable safety profile in young children. Erythromycin, clarithromycin, and azithromycin inhibit bacterial growth because these drugs block peptidyl tRNA dissociation from ribosomes and impair protein synthesis, which ultimately decreases the proliferation of *M. pneumoniae*. Doxycycline and minocycline also target ribosomal subunits and levofloxacin and moxifloxacin hinder DNA gyrase, which controls bacterial DNA replication and transcription. Dosages are recommended based on external factors and patient characteristics such as age, weight, and infection severity in children [18,19]. EM is managed with corticosteroids and IVIg to modulate severe inflammatory responses [20].

Prompt and accurate diagnosis is critical, with PCR testing being pivotal. Differential diagnosis should consider conditions like SJS, viral exanthems, and drug eruptions. Comprehensive evaluation using clinical history, serological tests, and, if necessary, skin biopsies ensure appropriate therapeutic intervention and optimal patient outcomes.

## Conclusions

Pediatric EM is an acute, immune-mediated condition affecting skin and mucosal surfaces. This case highlights an 11-year-old boy who developed high-grade fever, persistent oral ulcers, lip swelling, and a characteristic targetoid rash following an *M. pneumoniae* infection. Initially misdiagnosed, he was later treated with IV azithromycin, corticosteroids, and IVIg, leading to significant clinical improvement.

This case underscores the importance of early molecular diagnostics, particularly PCR testing, in differentiating *M. pneumoniae*-induced EM from other mucocutaneous conditions such as SJS, viral infections, and drug-induced reactions. Given the immune-mediated nature of this condition, a multidisciplinary approach - incorporating dermatologists, infectious disease specialists, and critical care teams - is essential for optimal management. Future research should focus on identifying biomarkers for disease severity and evaluating the efficacy of targeted immunomodulatory therapies in *M. pneumoniae*-induced EM. Establishing standardized treatment guidelines will help improve clinical outcomes, particularly in pediatric populations.
